# A framework for design optimization across multiple concepts

**DOI:** 10.1038/s41598-024-57468-2

**Published:** 2024-04-03

**Authors:** Angus Kenny, Tapabrata Ray, Hemant Singh

**Affiliations:** School of Engineering and Technology, University of New South Wales, Canberra, ACT 2600 Australia

**Keywords:** Mechanical engineering, Information technology, Aerospace engineering

## Abstract

In engineering design, there often exist multiple conceptual solutions to a given problem. Concept design and selection is the first phase of the design process that is estimated to affect up to 70% of the life cycle cost of a product. Currently, optimization methods are rarely used in this phase, since standard optimization methods inherently assume a fixed (given) concept; and undertaking a full-fledged optimization for each possible concept is untenable. In this paper, we aim to address this gap by developing a framework that searches for optimum solutions efficiently across multiple concepts, where each concept may be defined using a different number, or type, of variables (continuous, binary, discrete, categorical etc.). The proposed approach makes progressive data-driven decisions regarding which concept(s) and corresponding solution(s) should be evaluated over the course of search, so as to minimize the computational budget spent on less promising concepts, as well as ensuring that the search does not prematurely converge to a non-optimal concept. This is achieved through the use of a tree-structured Parzen estimator (TPE) based sampler in addition to Gaussian process (GP), and random forest (RF) regressors. Aside from extending the use of GP and RF to search across multiple concepts, this study highlights the previously unexplored benefits of TPE for design optimization. The performance of the approach is demonstrated using diverse case studies, including design of a cantilever beam, coronary stents, and lattice structures using a limited computational budget. We believe this contribution fills an important gap and capitalizes on the developments in the machine learning domain to support designers involved in concept-based design.

## Introduction

The engineering design process can be broadly thought of as comprising the following key stages^1^: problem definition, conceptual design, preliminary design and detailed design. As the name suggests, the first stage attempts to clearly assemble and transform the design requirements into quantifiable terms, including the objectives, constraints, specifications and other statutory norms. In the second phase, the designers draw up some plausible conceptual solutions to the problem. Such concepts, which are intended to satisfy the same design requirements, may look significantly different from each other, given the creative possibilities of applying scientific principles to achieve a desired outcome. Once a concept is selected, the solution is further refined through preliminary and detailed design that fleshes out all the finer components of the final product.

Evidently, concept design is a vital and a challenging step of this process as the final product configuration and performance rest heavily upon the decisions made in this phase^2^. By some estimates, decisions taken during the early phases, i.e., concept and preliminary design are known to affect up to 70% of the life cycle cost of the product^3^. The importance of exploring multiple competing concepts during early phases of design^1^ has long been advocated by designers, since early fixation on a single concept may lead to longer-term compromises. A number of practical case studies that deal with evaluating multiple concepts for a given design problem exist in the literature. For example, monohull and catamaran designs were evaluated as potential concepts for passenger transport^4^, three air induction system concepts were considered during the design of a power train^2^, various topologies were considered as concepts while designing truss structures^5^, and material choices and geometric variables were used to define various concepts for the design of rigid inflatable structures, compliant bicycle derailleurs and mobile phone battery connectors^6^. Noise absorption characteristics of various lattice structure configurations were also assessed during the phase of concept selection based on physical experiments^7^.

However, although the importance of an informed selection of promising concept(s) to carry forward in the design process is well-acknowledged, the task is far from trivial. It involves design space exploration of possible configurations corresponding to each concept to gauge its potential, while being conscious of cost involved in evaluating each candidate design. If too few design configurations belonging to a concept are evaluated in preliminary assessment, there is a possibility of the concept being rejected prematurely, without realizing its true potential. On the other hand, conducting an exhaustive design space exploration (through optimization or sampling) of each concept is often impractical since it may require significant computational, temporal or financial expense. This is particularly due to the numerical simulations (e.g. computational fluid dynamics, finite element analysis) or physical experiments (e.g. wind tunnel test, structural tests) that are commonly used in engineering for evaluating *each* candidate design^8^. Hence, the number of evaluations—that are practically affordable—are typically very limited.

**Figure 1:**
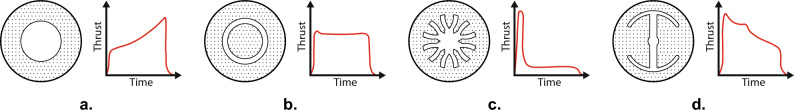
Different grain geometry profiles^9^ and their associated thrust profiles. Pictured are: (**a**) tubular; (**b**) rod-and-tube; (**c**) multi-fin; (**d**) double anchor.

On closer inspection, the above scenario can be thought of as a specialized type of optimization problem – one where the solution approach has to search *across multiple concepts* for the best possible design(s) and the associated concept(s) concurrently. Effectively, a method designed to solve such problems would need to efficiently explore the concepts in a manner such that the non-promising ones are filtered out systematically, while the most promising one(s) are given a higher chance to evolve to their optimum design configuration. Different concepts may have different numbers and types of design variables, which would pose significant challenges to a search method operating in this paradigm. As an example, consider the problem of designing the geometry of the propellant grain for a rocket^9^, illustrated in Fig. 1, where each shape (concept) is defined by its own variables: tube internal diameter is used to define tubular concepts (Fig. 1a), tube internal diameter and rod external diameter is used for rod and tube concepts (Fig. 1b), number of fins and their shape parameters are used for multi-fin designs (Fig. 1c) and several shape parameters are used to define double anchor designs (Fig. 1d). A number of other challenges may also be envisioned, including multiple objectives, the non-linearity of the underlying functions associated with a given concept and limited computing budget. Therefore, there can be disparities in the individual difficulties of optimizing each concept, which need to be considered, in order to appropriately allocate the computational budget.

Unfortunately, development of methods that can deal with such an optimization problem, i.e., spanning multiple concepts, has received scarce attention in the literature^10^. As evident from a wide body of literature in the field of design optimization, the approaches usually assume a fixed model, which makes them more suited to the later stages of the design; leading to lost opportunities to exploit their potential in early phases. That said, there have been some recent studies and growing interest in this domain^5,6,10–12^ which provide the key motivation to advance this line of query. These works have typically discussed the case of *multi-objective* multi-concept problems where the overall Pareto-optimal front might comprise solutions from one or more concepts. Some of the earlier studies^5^ highlight the idea of concept selection for multi-objective problems using the notion of s-Pareto frontiers coupled with normal constraint method. Others have discussed building more case studies^6,10^ and evaluate the performance of some evolutionary optimization approaches for multi-objective multi-concept problems^10,11,13,14^. Overall, we believe there remains a significant scope for development in this space. For example, somewhat counter-intuitively, while there has been some activity in the multi-objective domain, there are no single-objective multi-concept algorithms for this task. Moreover, the problems considered in the literature have been typically of a simple form, comprising only a few continuous variables. Last, but not least, the existing methods have not considered severe restrictions on the number of evaluations, except when testing some preliminary approaches and highlighting their limitations on multi-objective problems^10^.

To address some of the above gaps, in this work, we introduce an optimization approach that is specifically designed to solve problems encountered during concept design where: (a) there are a few possible design concepts each defined using its own set of concept specific variables; (b) the variables themselves can be continuous, discrete, integers or categorical; and (c) there is a practical limit on the number of design evaluations since computationally expensive simulations may be involved in the assessment of a design’s performance. Then, we demonstrate the performance of the proposed approach on three practical case studies, involving design of a cantilevered beam, coronary stent and lattice structures. We restrict this particular study to development of single-objective approaches, but the extension of this framework to handle multi-objective problems more efficiently is envisioned in the future work.

Related to single-objective problems in the multi-concept domain, certain well-known problem classes are also worth mentioning here, to clarify their differences from the type of problems targeted in this work. These include metameric optimization problems^15^ and automated machine learning based on pipeline optimization^16^. In metameric optimization problems, the solutions are represented using variable length chromosomes which have *repeated segments*. Some representative examples include design of wind farms, where the number of wind mills along with their location are considered as design variables, clustering problems where the ideal number and location of clusters are being sought^17^. The key aspect these problems differ from multi-concept problems is that for each analogous “concept” (e.g. number of wind farms/clusters), the solution space only changes by the addition of an identical set of variables (e.g. a set of co-ordinates); The “concepts” do not look structurally very different from each other in terms of types of variables and the physical quantities they represent. As for pipeline optimization, the structure of a pipeline, which is e.g., in the form of a decision tree or a graph, can be thought of as a concept, while the associated hyper-parameters become the concept-specific design variables. Pipeline optimization problems are commonly referred in the domain of machine learning as combined algorithm selection and hyperparameter (CASH) optimization problems^18^, which bear some similarity to the problem studied here. The parameters are often mixed in nature, including binary, continuous and discrete. However, in pipeline optimization the space of “concepts” themselves is unbounded, since unlimited combinations of underlying operators/data transformation techniques are possible. On the other hand, in multi-concept optimization, the number of potential concepts tend to be practically limited, as typically only a finite number of practically viable concepts exist to solve a given engineering design problem. More generally, CASH optimization problems could also be seen as a case of bilevel optimization^19^. In bilevel problems, an *upper level* optimization problem is solved, subject to finding optimal solutions of a nested *lower level* problem as its constraint.

To deal with the class of problems considered in this study, a few key challenges need to be overcome. These challenges are briefly outlined below to provide the rationale and considerations behind the algorithm design presented in the next section.

- *An efficient means to allocate computing budget across various concept search spaces:* Along this line, a common approach within an evolutionary framework is to use elitism in parent selection, which results in better performing concepts generating higher number of offspring^12,20,21^. A similar approach using another meta-heuristic, simulated annealing, utilized roulette wheel selection to assign higher preference to fitter concepts^17^. A second approach is known as successive halving, where the surviving population is reduced by half in every iteration, eliminating the worst half^22^. This scheme has been applied in machine learning to deal with pipeline optimization problems^22^.
- *An approximator that is capable of dealing with mixed variable types and can predict expected performance along with its uncertainty:* When the computational expense of evaluating a design is high, it becomes almost imperative to use approximation functions (also referred to as surrogate models, regressors or meta-models) to supplement the search. These models provide a means to pre-select more promising solutions, based on predicted mean and/or uncertainties before investing in their true evaluation. Gaussian process (GP) models and random forest (RF) models are suitable for the purpose. Among these, GP models are already widely used in design optimization studies. It is important to highlight however that to deal with categorical variables in their native form, GP models commonly require the use of one-hot encoding^23^. This increases the number of variables significantly, making it prone to scalability issues. In recent years, there have been attempts to use latent space based mapping along with modified kernels within GP models to deal with qualitative variables in addition to discrete and continuous variables^24^.
- *An efficient sampler and acquisition functions to explore the concept design spaces defined using different variable types:* In its most basic form, a sampler can simply be a random solution generation scheme. However, more efficient samplers usually involve an optimization process guided by an acquisition function. If a solution for a given concept is represented as a chromosome, standard evolutionary operators can be used to deal with the underlying optimization process. However, if the solution is represented in more elaborate forms, such as a tree, more specialized recombination operators might be required. The acquisition function can be simple or complicated, and can be derived in many ways. For example, the true evaluation of the design’s objective value; the predicted performance based on a surrogate model—e.g., believer; or, the predicted performance along with uncertainty considerations and information about the best known solution—e.g., expected improvement (EI)^25,26^. Believer- and EI-based schemes are regularly used in surrogate assisted optimization^27,28^. They have also been applied to problems with multiple search spaces and multi-objective considerations^10^. Tree structured Parzen estimator (TPE)-based samplers^29,30^ are often used in applications such as machine learning pipeline optimization, where solutions are commonly represented as trees. Since TPE-based samplers can intrinsically deal with mixed variables and tree-based representations, we believe they have significant potential to deal with optimization problems involving multiple concepts.

With the above considerations, we present the optimization framework in Section “Optimization framework” which incorporates GP and RF approximators, believer and EI acquisition functions, as well as a TPE-based sampler. The framework can be instantiated with five different combinations of the above selections, and numerical experiments and analysis are presented on three case studies in Section “Practical case studies” to analyze their behaviour. Section “Conclusions and future work” provides concluding remarks and outlines some future avenues of inquiry. For the sake of brevity, acquisition function/approximator combinations are abbreviated to, e.g., Believer (GP) or EI (RF), for a Gaussian process regressor using a believer-based acquisition function, or random forest regressor using an expected improvement-based acquisition function, respectively.

## Optimization framework

The proposed framework is outlined in Algorithm 1. It starts by generating a set of initial samples for each concept and evaluating them. It then follows a simple *fit-optimize-evaluate* loop, maintaining an archive of solutions which have been evaluated so far. With each iteration, a regressor is built based on the truly evaluated samples available in the archive, and then the most promising sampling location is identified via maximization of an acquisition function for each concept. These suggested samples are then compared across concepts and the best over-all is selected for evaluation. This conforms to a steady-state paradigm (one true evaluation in each iteration) that is particularly suitable for scenarios with limited computing budget^8^. In case the selected sample already exists in the archive, a new sample from the same concept is generated randomly, evaluated and added to the archive to avoid redundant evaluation and promote search space exploration.

The framework includes a TPE-based sampler in addition to GP and RF regressors with believer- and EI-based acquisition functions for sampling. TPE is particularly attractive during concept-based design optimization as it represents the solutions/designs as trees and its sampling is analogous to EI. To the best of our knowledge, TPE has not been used for concept-based design optimization, though it has been known in the field of machine learning. GP and RF regressors have long been used in the context of surrogate-assisted optimization, with believer- and EI-based acquisition functions. Hence, they have been included in this framework to provide a baseline for the experimental results. As indicated earlier, to handle categorical variables in GP, one-hot encoding is often used to convert each category into a numerical (binary) vector. RF or TPE models do not require such special treatment as solutions are represented as trees instead of vectors.

In Algorithm 1, sample$(c,n)$ is a function which returns *n* random samples belonging to concept *c*. The samples are generated using Latin hyper-cube sampling and cast to their appropriate values and types. The procedures initialize, fit and optimize are generic procedures for any model type *T*. A more detailed description of these procedures and their implementation in the proposed framework is presented below.

**Algorithm 1:**
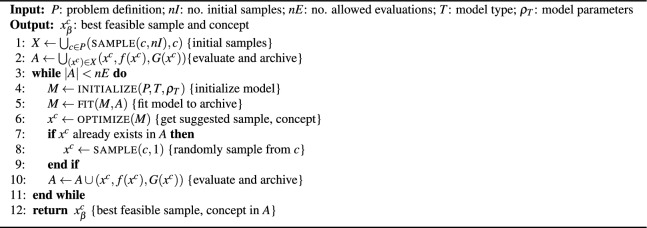
Proposed optimization framework

### Believer/EI-based samplers for GP and RF regressors

In computationally expensive optimization, sampling is typically guided using an acquisition function. An acquisition function refers to an auxiliary, cheap-to-evaluate function that is optimized in order to identify promising regions of the search space to sample. While a number of acquisition functions have been suggested in the literature, believer and EI are arguably the most widely used. The believer function simply queries the GP or the RF regressor for a given sample, and then “believes” that the approximation is accurate. This effectively translates to optimizing the predicted mean based on GP/RF model directly to identify the sample for the next true (expensive) evaluation. The EI acquisition function on the other hand uses the mean and standard deviation of the predicted performance based on the regressor along with the information of the best evaluated sample to define the fitness of the sample. The functional form of EI promotes sampling either in a location where the objective value is most likely to improve, or in a location where there is high uncertainty (i.e., under-sampled regions); making it more exploratory and less greedy compared to believer function.

Separate GP or RF regressors are instantiated for the objective function and each of the individual constraints for every concept. When fitting such regressors, all previously evaluated samples for each concept in the archive are used. Having initialized the regressors for each concept, the next step is to choose an acquisition function (believer or EI) and conduct a search to identify the best sampling location for each concept. An evolutionary algorithm (EA) is used to conduct this search. The EA with the believer acquisition function conducts the search using the predicted values of the objective and constraints. For the EI-based acquisition function, the samples are sorted lexicographically, by probability of feasibility, followed by the number of constraints satisfied and finally the sum of constraint violations. For the EA, the fitness metric for each sample is its lexicographically sorted rank, multiplied by its expected improvement in the objective function value. Algorithm 2 outlines the pseudo-code for these three procedures.

**Algorithm 2:**
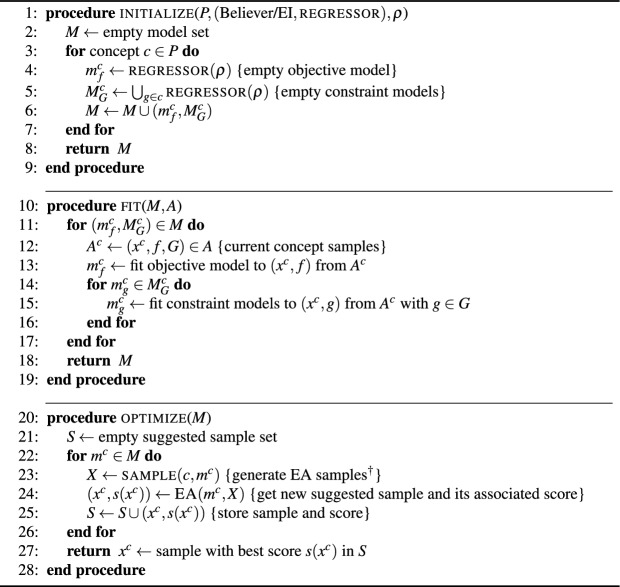
Believer/EI model

Here, *T* in Algorithm 1, which is passed to initialize is a 2-tuple: the first element specifies the acquisition function (believer or EI) and the second specifies the regressor model type (GP or RF) used with it. regressor$(\rho )$ is a generic function which creates an empty regressor model with the parameters in $\rho$. The function $\text {{EA}}(m,X)$ conducts a search based on an EA on model *m* with starting population *X* and returns the best sample within the allowed budget, along with its associated score.

### Multivariate TPE-based sampler

Once the initial samples are generated, evaluated and stored in the archive, individual TPE samplers are constructed for each concept. To construct a TPE sampler, the archived samples are first globally ranked with all feasible solutions ranked higher than all infeasible ones across all concepts. As TPE samplers use a single metric to determine the fitness of potential candidates, they cannot explicitly handle constraints or constraint violations. This metric is often defined as the ratio of two functions *l*(*x*)/*g*(*x*), where the function *l*(*x*) represents the likelihood of sample *x* being considered “good” (i.e., within a certain fitness threshold), and the function *g*(*x*) represents the probability density function used to model the data. Ranking the samples first by feasibility and then objective function ensures that when the *l*(*x*)/*g*(*x*) metric is maximized, the sampler yields a candidate that is the best for each concept, prioritizing feasible solutions over infeasible ones. Use of global ranks allow comparison across concepts, while still maintaining separate TPE samplers for each concept. Once the sampler is constructed, a number of candidates are generated and their fitness predicted. The sample candidate with the best fitness over all is selected for evaluation. We use multivariate TPE instead of independent TPE sampler as it allows a single sampler to be maintained per concept, as opposed to one per decision variable. In addition, multivariate TPE is more adept at dealing with problems where there is interaction between the variables.

In order to ensure a fair comparison between the approaches, i.e., GP and RF regressors with believer and EI based sampling and the multivariate TPE (MvTPE) based sampling, the number of queries from each method need to be equivalent. Hence the number of TPE candidates for query is set as the population size, multiplied by the number of generations used by the evolutionary algorithm, to maximize the acquisition functions discussed in the previous section. The pseudo-code of the three procedures i.e., initialize, fit and optimize for the multivariate TPE sampler is presented in Algorithm 3, where $\text {{TPE}}(\rho )$ is a function which creates an empty TPE model with the parameters in $\rho$; and, suggest$(m)$ is a function which queries TPE model *m* for a suggested sample, along with its associated score.

**Algorithm 3:**
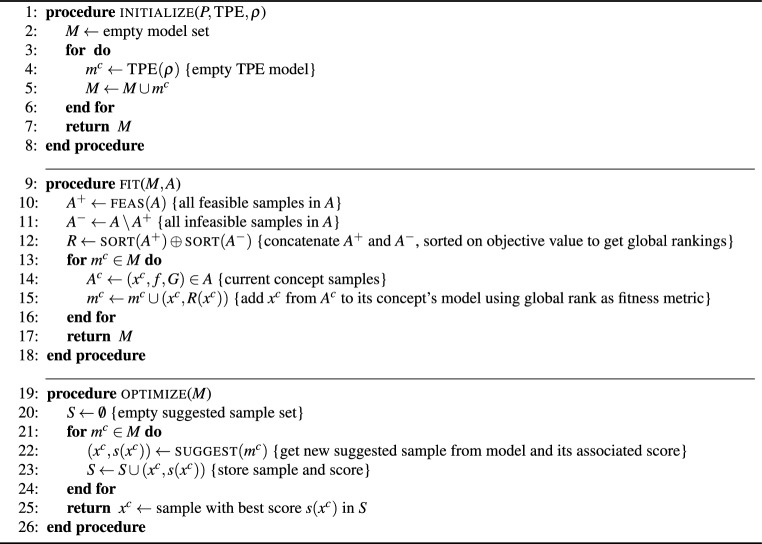
Multivariate TPE sampler

## Practical case studies

In order to illustrate the behavior of the approaches described in Section “Optimization framework”, three practical case studies are modeled and studied. They include design of a cantilevered beam, coronary stent and lattice structure. The details of the models, numerical experiments and related discussion is presented below in the respective sub-sections.

### Design of cantilevered beam

**Figure 2:**
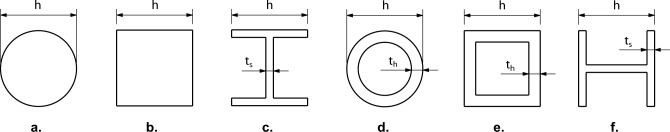
Beam cross-section geometry concepts. Pictured are: (**a**) Circular, (**b**) Square, (**c**) ISection, (**d**) CircularHollow, (**e**) SquareHollow, (**f**) HSection.

A cantilevered beam is a fundamental structural element that is used in a range of designs such as buildings, bridges and aeroplanes. Two simple numerical examples were adapted from the beam deflection problem presented elsewhere^24^. The two variants are referred to here as Beam-1 and Beam-2. Both the problems aim to minimize the mass of a cantilevered beam, subject to a deflection constraint when a downward force is applied at its end, described by Eq. (1). The beam has a fixed length and can have one of six different cross-sectional geometries, considered here as different concepts: circular, square, I-section, hollow circular, hollow square, H-section—for consistency these concepts have been given the labels Circular, Square, ISection, CircularHollow, SquareHollow and HSection, respectively. Each concept is controlled by a single decision variable *h* which is related to both the width and height of the section (Fig. 2).

Both Beam-1 and Beam-2 have the formulation:

1 $$\begin{aligned} \text {minimize} \quad AL\rho ; \quad \text {subject to} \quad&\frac{PL^3}{3EI} \le \delta _{max}, \end{aligned}$$

where, *A* is the cross-sectional area of the beam, $L=400$ mm is the length of the beam, $\rho$ is the density of the beam material, $P=600$ N is the force applied, *E* is the Young’s modulus of the beam material, *I* is the moment of inertia for the cross-section and $\delta _{max} = 5$ mm is the maximum allowed deflection under load. The cross-sectional area and moment of inertia for each of the concepts is given in Table 1.

In the case of Beam-1, the beam material is fixed as steel which has a density of $\rho = 7820 \times 10^9$ kg/mm^3^ and a Young’s modulus of $E=$ 200 GPa. For Beam-2, the material is considered as an additional categorical variable, with four possible values: steel, aluminium, carbon-fibre reinforced polymer (CFRP) and brass. The properties of these materials are presented in Table 2.

**Table 1:** Cross-sectional area and moments of inertia for the six beam cross-section concepts. Thickness of I- and H-sections is $t_s = 0.1h$ and wall thickness of hollow sections is $t_h = 0.15h$.

Concept label	Cross-sectional area, *A* [mm^2^]	Moment of inertia, *I* [mm^4^]
Circular	$\dfrac{\pi h^2}{4}$	$\dfrac{\pi h^4}{64}$
Square	$h^2$	$\dfrac{h^4}{12}$
ISection	$(3-2t_s)t_sh^2$ $\dfrac{1}{12}\left( t_sh((1-2t_s)h)^3 + 2\left( h^4t_s^3 + 3t_s(1-t_s)^2\right) \right)$	
CircularHollow	$\dfrac{\pi (h^2 - ((1-2t_h)h)^2)}{4}$	$\dfrac{\pi (h^4 - ((1-2t_h)h)^4)}{64}$
SquareHollow	$h^2 - ((1-2t_h)h)^2$	$\dfrac{1}{12}\left( h^4 - ((1-2t_h)h)^4\right)$
HSection	$(3-2t_s)t_sh^2$	$\dfrac{1}{12}\left( (1-2t_s)h^4t_s^3 + 2(t_sh^4)\right)$

**Table 2:** Beam material properties used in Beam-2.

Material	Density, $\rho$ (kg/mm^3^)	Young’s modulus, *E* (N/mm^2^)
Steel	7820e−09	200e+03
Aluminium	2700e−09	69e+03
CFRP	1900e−09	117e+03
Brass	8600e−09	100e+03

For benchmarking, the “ground truth” for each combination of beam cross-sectional geometry and material was established using the minimize method from the SciPy library with non-linear constraints. Each run started from a different random point, returning the minimum feasible mass after a maximum of 100,000 iterations. The best result obtained after 100 runs for each geometry and material combination is listed in Table 3, along with its corresponding *h* value.

**Table 3:** Ground truth for beam cross-sectional geometry and material combinations.

	Steel	Aluminium	CFRP	Brass
Circular				
$h^*$	2.2599e + 01	2.9488e + 01	2.5840e + 01	2.6874e + 01
$f(h^*)$	1.2547e + 00	7.3760e − 01	3.9860e − 01	1.9513e + 00
Square				
$h^*$	1.9798e + 01	2.5832e + 01	2.2637e + 01	2.3544e + 01
$f(h^*)$	1.2262e + 00	7.2084e − 01	3.8954e − 01	1.9068e + 00
ISection				
$h^*$	**2.3103e + 01** ^†^	3.0146e + 01	**2.6419e + 01** ^‡^	2.7477e + 01
$f(h^*)$	**4.6760e − 01** ^†^	2.7504e − 01	**1.4875e − 01** ^‡^	7.2724e − 01
CircularHollow				
$h^*$	2.3054e + 01	3.0078e + 01	2.6359e + 01	2.7415e + 01
$f(h^*)$	6.6597e − 01	3.9160e − 01	2.1179e − 01	1.0356e + 00
SquareHollow				
$h^*$	2.1206e + 01	2.7668e + 01	2.4247e + 01	2.5217e + 01
$f(h^*)$	7.1750e − 01	4.2181e − 01	2.2817e − 01	1.1156e + 00
HSection				
$h^*$	2.9574e + 01	3.8589e + 01	3.3818e + 01	3.5170e + 01
$f(h^*)$	7.6626e − 01	4.5042e − 01	2.4371e − 01	1.1916e + 00

Highlighted, are best results for Beam-1 ($\dagger$) and Beam-2 ($\ddagger$).

Each of the five approaches—Believer (GP/RF), EI (GP/RF) and MvTPE—were executed on Beam-1 and Beam-2. To observe their statistical behaviour, each algorithm was run on each problem 21 times with a different random seed (starting population). The number of initial evaluations (*nI* in Algorithm 1) were set at $nI = 11D_{sum}+1$, where $D_{sum}$ is the sum of the number of variables, across all concepts. In the case of Beam-1, the material is fixed and the thicknesses ($t_h$ and $t_s$) are a constant ratio of *h*, so each of the six concepts is defined by a single variable, meaning $D_{sum}$ is 6, and $nI=11 \times 6 + 1 = 67$. Beam-2 is similar to Beam-1, but there is an extra decision variable determining the material for each concept, so $nI = 11 \times 12 + 1 = 133$. The total computing budget (*nE* in Algorithm 1) was set as $nE = 100D_{max}$ evaluations, where $D_{max}$ is the maximum number of variables for a single concept ( $nE = 100$ for Beam-1 and $nE=200$ for Beam-2). The believer and EI acquisition functions were optimized using non-domination sorting genetic algorithm II (NSGA-II), as implemented in the Pymoo Python library^31^ with their default settings. Note that for single-objective optimization of an acquisition function, non-dominated sorting defaults to simply ascending sort (for minimization) or descending sort (for maximization). To stay consistent with the believer and EI models, the MvTPE sampler selected from a pool 10,000 candidates. The results of these experiments are presented in Table 4.

**Table 4:** Experimental results for believer, expected improvement and multivariate TPE models on two variants of the beam cross-section deflection problem.

	Believer		EI		MvTPE
	GP	RF	GP	RF	
Beam-1					
${h_m}^*$	2.3108e + 01	**2.3103e + 01**	2.3946e + 01	2.5737e + 01	2.3738e + 01
$f({h_m}^*)$	4.6768e − 01	**4.6746e − 01**	5.0223e − 01	5.8014e − 01	4.9351e − 01
$f({h_\mu }^*)$	4.8319e − 01	**4.6746e − 01**	5.0869e − 01	5.6934e − 01	5.0464e − 01
Std. dev	3.7176e − 02	8.2479e − 07	3.7216e − 02	5.3902e − 02	3.4455e − 02
Concept	ISection	ISection	ISection	ISection	ISection
Beam-2					
${h_m}^*$	2.8077e + 01	**2.6431e + 01**	2.8417e + 01	2.8903e + 01	2.6817e + 01
$f({h_m}^*)$	1.6775e − 01	**1.4866e − 01**	1.7184e − 01	1.7777e − 01	1.5304e − 01
$f({h_\mu }^*)$	1.7541e − 01	1.7211e − 01	1.8653e − 01	1.9173e − 01	**1.5656e − 01**
Std. dev	2.4301e − 02	4.0869e − 02	3.6234e − 02	3.8003e − 02	9.7433e − 03
Concept	ISection	ISection	ISection	ISection	ISection
Material	CFRP	CFRP	CFRP	CFRP	CFRP

Given are the median resultant beam geometry variable ${h^*}_m$, its corresponding objective $f({h^*}_m)$, the mean objective $f({h^*}_\mu )$and its standard deviation, along with the best concept (and material) for each problem variant and model type, over 21 runs. Best values obtained among the compared algorithms are shown in bold.

As Beam-1 is a simple problem with a single objective and a single, continuous variable, it is expected that the believer model should perform well; compared to the EI and MvTPE models, which have uncertainty considerations. Analyzing the results in Tables 3 and 4, it can be seen that this is indeed the case, with Believer (RF) achieving the best median and mean results across 21 runs, with Believer (GP) a close second. These results corroborate well with the ground truth in Table 3.

In the case of Beam-2, the extra categorical variable for material choice introduces additional complexity. In this instance, Believer (RF) still produced the best median result, however MvTPE produced a better mean result over all. Since the GP based regressor uses one-hot encoding, it had to deal with a problem with significantly larger number of variables since an extra *N* variables are added to the problem for every categorical variable with *N* categories. This significantly increases the size of the search space and, hence, is likely to adversely affect performance—as can be seen in the results for Believer (GP) on Beam-2. In contrast, RF regressors and MvTPE can natively process categorical variables, which is likely to lead to better performance; supported by the experimental results.

While Table 4 suggests ISection as the best cross-sectional geometry concept, the results in Table 3 show that the CircularHollow, SquareHollow and HSection concepts are still reasonably competitive, and far better than Circular and Square. Although the selection of these concepts is unlikely to lead to globally optimal solutions, it is still possible that they could produce competitive designs. Table 5 presents the results for the median MvTPE run on Beam-2. We also include the results obtained by other approaches for this seed (same initial set of solutions have been used by all approaches). It can be seen that Believer (RF) and EI (GP) both conclude that CircularHollow is the best concept to choose.

**Table 5:** Detailed results for the median MvTPE run from 21 runs on Beam-2, across all models.

	Believer		EI		MvTPE
	GP	RF	GP	RF	
$h^*$	2.7735e + 01	2.6635e + 01	2.7065e + 01	3.3200e + 01	**2.6817e + 01**
$f(h^*)$	1.6369e − 01	2.1596e − 01	2.2299e − 01	2.3456e − 01	**1.5304e − 01**
Concept	ISection	CircularHollow	CircularHollow	ISection	ISection
Material	CFRP	CFRP	CFRP	CFRP	CFRP

Given are the resultant beam geometry variable $h^*$, its corresponding objective $f(h^*)$ along with the best concept and material for that run. Best values obtained among the compared algorithms are shown in bold.

**Figure 3:**
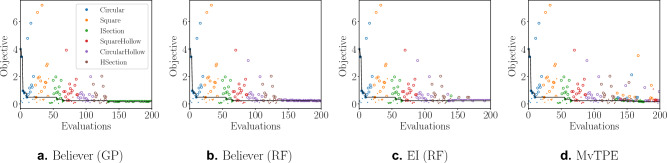
Comparison plots for the median run of MvTPE on Beam-2. Circles indicate feasible solutions and small dots indicate infeasible ones. A circle is filled if it represents a solution improving on all prior to it.

The plots in Fig. 3 illustrate this comparison further. All four plots show the first 133 evaluations comprising the same set of initial solutions, with the remaining 67 reserved for the main search. Figure 3a,b demonstrate the greedy nature of the believer-based approach, with both “locking-on” to a single concept and rarely straying from it for the entirety of the search. This has not been detrimental for Believer (GP), which chooses the correct concept and sticks with it. This greedy behavior however has been problematic for Believer (RF), since it incorrectly identified the concept from the outset and was trapped in a local optimum. Figure 3c shows the EI (RF) search to be slightly more diverse, however it struggles to find many feasible solutions. In contrast, the MvTPE search in Fig. 3d produces sufficiently diverse, good quality feasible solutions across many different concepts. This diversity in the search suggests that the MvTPE approach may be effective in avoiding local optima, while also identifying a range of competitive solutions.

### Coronary stent design

**Figure 4:**
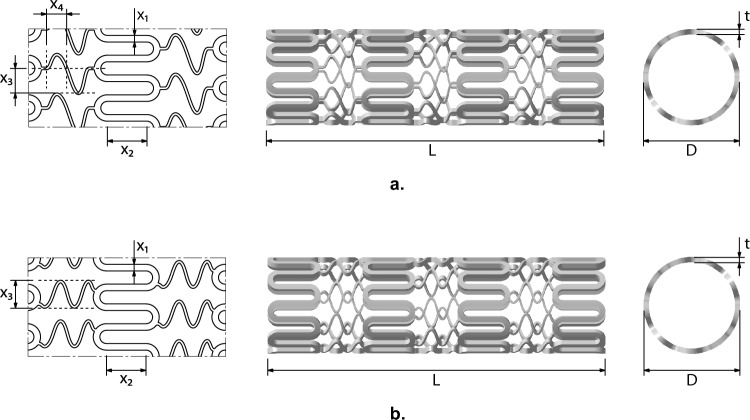
Stents used in this study. Left figures give the schematic diagram with variable labels; middle and right illustrate the stent in its “rolled-up” state.

**Figure 5:**
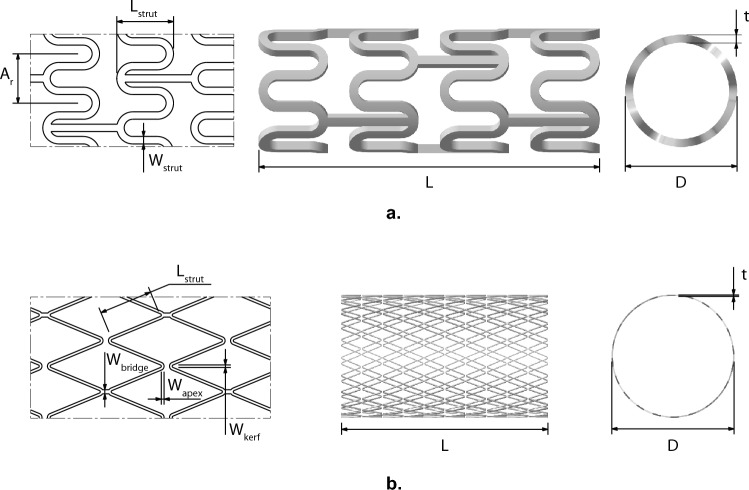
Other stents examples, not used in this study. Left figures give the schematic diagram with variable labels (as defined in their respective publications); middle and right illustrate the stent in its “rolled-up” state.

Coronary stents are essentially wire mesh scaffolds that are deployed to re-open narrowed coronary arteries^32^. While the stents have the same common purpose they may look significantly different from each other. Four such designs are presented in Figs. 4 and 5. Of these, we use two of the stents (Stent-A, Stent-B) shown in Fig. 4 for the study herein. The data corresponding to these designs is obtained from two previously published papers^32,33^.

The decision variables are represented with a vector whose length is determined by the specific design concept in question.

Stent-A is described using four continuous variables ($x_1,x_2,x_3,x_4$, corresponding to $W_{strut}$, $h_c$, $p_1$ and $p_2$, respectively) while Stent-B is described using three continuous variables ($x_1,x_2,x_3$, corresponding to $W_{strut}$, $h_c$ and $n_{height}$, respectively). The original study for Stent-A considered four responses: recoil *R*, volume added stress *VAS*, volume average drug (inverse) *VAD*^-1^ and flexibility metric *FM*; while the original study for Stent-B considered six: recoil *R*, volume added stress *VAS*, haemodynamic low and reverse flow index *HLRFI*, volume average drug *VAD*, uniformity of drug distribution $D_{dev}$ and flexibility metric *FM*. For this investigation, we consider the common responses: *VAS*, *VAD*^-1^, *FM* and *R*.

**Figure 6:**
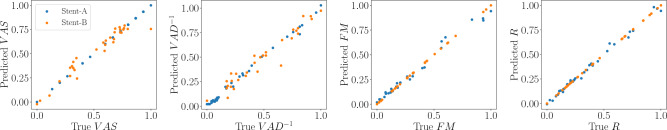
Scatter plots showing normalized true observed responses versus predicted responses obtained from the selected models, for each stent design configuration in the reported data.

Coronary stent design configurations can be very computationally expensive to evaluate; often requiring complex finite element analysis simulations, along with computational fluid dynamics simulations of haemodynamics and drug distribution^33^. Due to the intensive computational resources and time required for these evaluations, there is limited data available for each design concept. To address this challenge and enable our algorithm to evaluate design configurations for which no reported data are available, we have employed regression models to represent each response for the reported design configurations. In order to model the responses for each design configuration as accurately as possible, we tested six regression models from the scikit-learn Python library^34^: Ridge, Lasso, ElasticNet, PolynomialRegressor, RandomForestRegressor, and GradientBoostingRegressor. The hyperparameters for each model were tuned using a grid-search approach to ensure optimal performance. To assess the effectiveness of each model, we employed a leave-one-out cross-validation technique, with negative mean squared error as the evaluation metric. This process enabled the best model and hyperparameter configuration to be identified for each individual response, tailored to each design concept. Table 6 provides the results of this model selection process, and Fig. 6 demonstrates the prediction accuracy of the models for each response on the original reported data. In this figure, the normalized prediction of each selected regression model is plotted against the normalized true response, for each of the reported design configurations. While the models do not replicate the original data with exact precision, the purpose of this study is to demonstrate the ability of our proposed method to effectively optimize across multiple design concepts, not to improve state-of-the-art coronary stent design. In this context, the models adequately serve as the de facto “ground truth” for the stent design configuration responses.

**Table 6:** Leave-one-out cross-validation error obtained from best hyperparameter combination found, for each regression model on each response.

	Stent-A				Stent-B			
Model	*VAS*	$VAD^{-1}$	*FM*	*R*	*VAS*	$VAD^{-1}$	*FM*	*R*
Ridge	5.9930e − 06	2.0652e − 03	7.1052e − 02	3.8066e − 04	4.7410e − 06	4.1339e − 05	1.5167e − 03	2.7324e − 04
Lasso	5.5590e − 06	2.0716e − 03	7.1514e − 02	3.6010e − 04	4.7721e − 06	4.1532e − 05	1.5297e − 03	2.7455e − 04
ElasticNet	5.0481e − 06	2.0713e − 03	7.1349e − 02	3.5034e − 04	4.7502e − 06	4.1069e − 05	1.5293e − 03	2.7448e − 04
PolynomialRegressor	**3.8060e − 07**	**1.9039e − 04**	2.9520e − 02	**1.3410e − 04**	**2.9483e − 06**	**3.0020e − 05**	1.4372e − 03	2.2918e − 04
RandomForestRegressor	1.4191e − 06	8.7346e − 04	**2.8396e − 02**	5.0322e − 04	3.4167e − 06	4.1822e − 05	9.5205e − 04	2.9900e − 04
GradientBoostingRegressor	1.7809e − 06	8.3799e − 04	3.5441e − 02	3.8587e − 04	3.5834e − 06	5.2058e − 05	**8.2249e − 04**	**2.2201e − 04**

Bold indicates model was selected for response ground truth.

**Figure 7:**
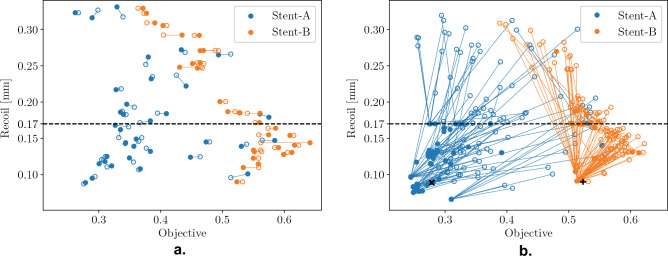
Objective and recoil constraint plots. In the left figure, filled circles represent the reported stent design configuration, and their responses, for Stent-A^32^ and Stent-B^33^. Unfilled circles represent the predicted responses of the regression models on the same inputs. In the right figure, unfilled circles represent initial, randomly sampled stent design configurations. Filled circles represent the result after optimizing each sample with the SLSQP algorithm. Symbols $\varvec{\times }$ and $\varvec{+}$ represent the best reported configurations for Stent-A and Stent-B, respectively. In both figures, the dashed line indicates maximum recoil value $R=0.17$ mm, solutions above this line are considered infeasible.

This claim is supported by the plots in Fig. 7. To transform this problem, which considers four response metrics, into a single-objective, constrained optimization problem. We utilize the normalized average of the *VAS*, *VAD*^-1^, and *FM* responses, as the combined objective to minimize. The optimization is subject to constraint on recoil value, *R*, to not exceed 0.17 mm. Figure 7a displays the responses for the design configurations of Stent-A and Stent-B, when formulated in this manner. In this plot, filled circles represent the actual combined objective and recoil constraint values of the design configurations, while unfilled circles denote the combined objective and recoil constraint values predicted by the selected regression models. The proximity of most predicted points to their actual counterparts suggests that the regression models provide an adequate representation of the data. To further validate the selected regression models and the problem formulation itself, the sequential least squares programming (SLSQP) algorithm from the Scipy Python library^35^ was employed. Using the reported data to establish variable bounds, 100 initial design configurations for each stent concept were generated through random sampling. These configurations were optimized for a maximum of 10,000 function evaluations, with the predicted recoil response serving as a non-linear constraint. Figure 7b visualizes this optimization process, where unfilled circles signify the initial samples, and filled circles illustrate the outcomes of applying the SLSQP algorithm. Symbols $\varvec{\times }$ and $\varvec{+}$ highlight the best configurations for Stent-A and Stent-B as per the reported data, respectively. This visualization reveals that, regardless of the starting points, the optimization trajectory consistently gravitates towards these regions. Furthermore, the distribution of the sampled points for each concept mirrors the distribution of points of the reported data in Fig. 7a, with the points for Stent-A exhibiting a broader spread compared to Stent-B, which are confined to a narrow band. Both plots collectively underscore Stent-A’s superior performance over Stent-B—in the proposed problem formulation. Table 7 presents the best feasible solutions for each concept as identified in the reported data alongside those derived from the optimized regression models.

**Figure 8:**
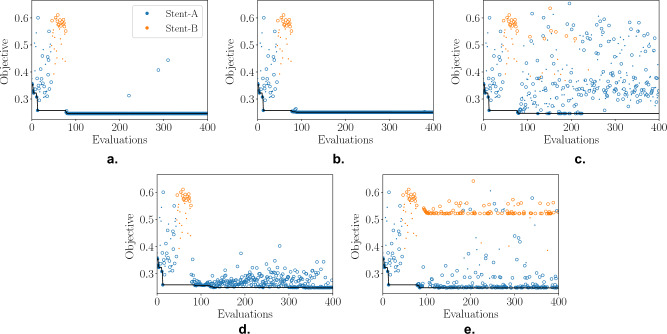
Comparison plots for the median run of MvTPE on StentOpt. Circles indicate feasible solutions and small dots indicate infeasible ones. A circle is filled if it represents a solution improving on all prior to it.

**Table 7:** Responses of best feasible solution from reported data and after optimizing the regression models.

	Reported		Optimized model	
	Recoil (mm)	$f(x^*)$	Recoil (mm)	$f(x^*)$
Stent-A	8.9000e − 02	2.7845e − 01	9.6374e − 02	2.4473e − 01
Stent-B	9.0100e − 02	5.2329e − 01	1.0438e − 01	5.1099e − 01

**Table 8:** Experimental results for believer, expected improvement and multivariate TPE models on the stent optimization problem.

	Believer		EI		MvTPE
	GP	RF	GP	RF	
$f({x_b}^*)$	2.4473e − 01	2.4574e − 01	2.4473e − 01	2.4511e − 01	2.4531e − 01
$f({x_m}^*)$	2.4473e − 01	2.7142e − 01	2.5184e − 01	2.5092e − 01	2.4967e − 01
$f({x_\mu }^*)$	2.4487e − 01	2.7002e − 01	2.5257e − 01	2.5319e − 01	2.5199e − 01
Std. dev	2.6376e − 07	1.2370e − 02	6.4119e − 03	7.0673e − 03	8.2140e − 03
Concept	Stent-A	Stent-A	Stent-A	Stent-A	Stent-A

Given are best $f({x^*}_b)$, median $f({x^*}_m)$ and mean $f({x^*}_\mu )$ objective values, along with the standard deviation and best concept, over 21 runs.

Having formulated the problem, the Believer (GP/RF), EI (GP/RF) and MvTPE approaches were applied. Table 8 presents the statistics of the results obtained across multiple (21) runs. All approaches managed to identify Stent-A as the best choice of concept, which is supported by the original data and its regression models. Although there is not a lot to choose between the different methods in terms of solution quality, Fig. 8 illustrates the difference between them when it comes to the breadth of search. This figure presents the convergence plots of all five methods when applied to this problem using the same initial random seed that produced the median MvTPE run. After the initial random samples were generated, both the believer- and EI-based models almost exclusively selected Stent-A samples for evaluation, with EI (GP) being the only one to evaluate any additional Stent-B samples (Fig. 8c). Although the plots in Fig. 8 do not give any explicit information about where in the decision space the samples are drawn from, it can be inferred by the low variation in objective value that the believer-based models (Fig. 8a,b) searched in a narrow region; this is not surprising as Fig. 7b demonstrated that there is only a single basin of attraction. The EI-based models (Fig. 8c,d) were more exploratory, however they only really explored the region of the search space associated with the Stent-A concept. In contrast, MvTPE (Fig. 8e) sampled liberally from both concepts, while ensuring good solution quality for both. These observations are consistent with those made previously.

### Lattice structures

The final example is another practical problem, adapted from a previously reported study^10^, involving the design of three-dimensional lattice structures. Broadly speaking, a lattice is a class of cellular material consisting of any repeatable structure that fills space. There are typically three main classes of lattice structures: surface-based lattices, where the shape, size and density of the structure is controlled by trigonometric equations; strut-based lattices, where rod-like structures are connected in various orientations to form unit cells, either stochastically (e.g., foams) or periodically (e.g., crystals); and planar-based lattices, where a periodic, two-dimensional pattern is extruded along a third axis (e.g., a honeycomb). The lightweight nature and excellent thermal and mechanical properties of lattice-based materials makes them ideally suited for a wide range of engineering applications such as heat exchangers, biomechanical implants, and acoustic panels.

**Figure 9:**
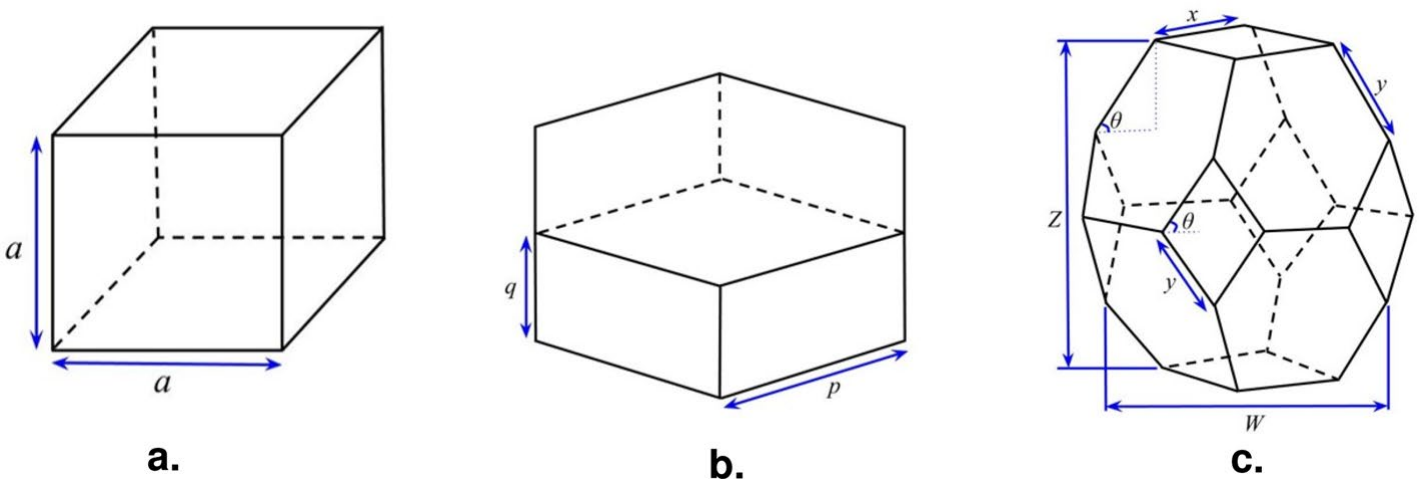
Unit cell lattice structure concepts.

The lattice structures investigated in this study are strut-based lattices, formed by the periodic, three-dimensional arrangement of regular, polyhedral unit cells. Three concepts are considered for the unit cells: cube, hexagonal prism and elongated tetrakaidecahedron—given the labels Cube, Hex and Tkdh, respectively. The geometries of these unit cells are encoded as a vector of variables, with length of two, three or four, depending on the concept. This includes an additional variable for the strut radius ($r_c$, $r_h$, $r_t$, respectively). All three polyhedra are categorized as *parallelohedra*, which allows the formation of lattice structures through unit cell translation, without rotation. Figure 9 illustrates the three concepts and their associated variables. For further discussion on lattices and a detailed definition of the original problem, see^10^.

In its original form, this lattice-structure design problem involves the minimization of volume of material within a fixed bounding box, the maximum displacement under load, and the maximization of surface area. However, we have modified this multi-objective problem into three, constrained single objective ones referred to as Lattice-1, Lattice-2 and Lattice-3 (Eqs. 2, 3 and 4, respectively).

2 $$\begin{aligned} \text {Given} \quad&\varvec{z}_c = (a,r_c),\ \varvec{z}_h = (p,q,r_h),\ \varvec{z}_t = (x,y,\theta ,r_t):\nonumber \\ \text {minimize} \quad&V(\varvec{z}), \text { subject to} \quad \delta (\varvec{z}) \le \delta _{max}; \quad 0 \le \frac{x}{y} \le 2\sqrt{2}; \quad \arcsin {\left\{ \frac{\sqrt{2x}}{5y}+ \frac{\sqrt{2}}{10}\sqrt{10-\frac{x^2}{y^2}}\right\} } \le \theta \le \frac{\pi }{2};\end{aligned}$$

3 $$\begin{aligned} \text {minimize} \quad&V(\varvec{z}), \text { subject to} \quad 0 \le \frac{x}{y} \le 2\sqrt{2}; \quad \arcsin {\left\{ \frac{\sqrt{2x}}{5y}+ \frac{\sqrt{2}}{10}\sqrt{10-\frac{x^2}{y^2}}\right\} } \le \theta \le \frac{\pi }{2};\end{aligned}$$

4 $$\begin{aligned} \text {minimize} \quad&\delta (\varvec{z}), \text { subject to} \quad 0 \le \frac{x}{y} \le 2\sqrt{2};\nonumber \\&\quad \arcsin {\left\{ \frac{\sqrt{2x}}{5y}+ \frac{\sqrt{2}}{10}\sqrt{10-\frac{x^2}{y^2}}\right\} } \le \theta \le \frac{\pi }{2}. \end{aligned}$$

Here, $V(\varvec{z})$ is the volume of the lattice represented by the vector $\varvec{z}$ and $\delta (\varvec{z})$ is its displacement under load. All three formulations share the same variable bounds:

$$\begin{aligned} a&\in [10,20]; \quad p \in \left[ \frac{10}{2\cos {30^\circ }},\frac{20}{2\cos {30^\circ }}\right] ; \quad q \in [10,20]; \quad x \in \left[ \frac{10-5\sin {55^\circ }\cos {65^\circ }}{\sqrt{2}},\frac{20-5\sin {55^\circ }\cos {65^\circ }}{\sqrt{2}}\right] ;\\ y&\in \left[ \frac{10}{4 \sin {55^\circ }},\frac{20}{4 \sin {55^\circ }}\right] ; \quad \theta \in [55^\circ ,65^\circ ];\quad r_c \in [0.5,1]; \quad r_h\in [0.5,1]; \quad r_t\in [0.5,1]. \end{aligned}$$

Lattice-1 minimizes the volume of the lattice structure, while setting a constraint on the maximum displacement; Lattice-2 minimizes the volume of the lattice structure with no constraint on the displacement; and Lattice-3 minimizes the displacement of the lattice with no other constraints at all.

**Figure 10:**
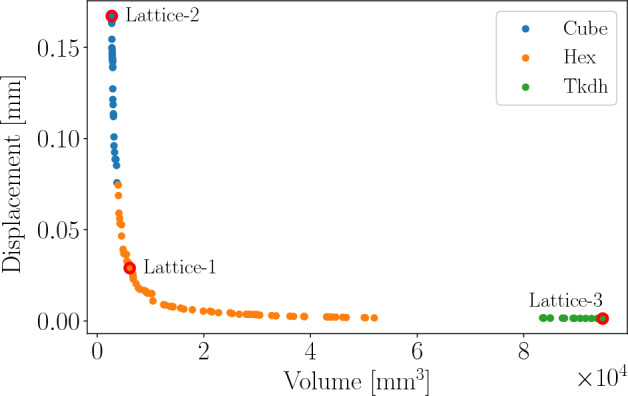
Ground truth for lattice structures problem, as reported in Parker et al.^10^. Circled in red are the optima for the three problem variants: Lattice-1, Lattice-2 and Lattice-3.

As this problem is intractable to exact, analytical methods, finite element analysis (FEA) is performed to evaluate the deflection of any given design. Due to the resource-intensive nature of the FEA simulations, the reported results from^10^ are used as the ground truth for comparison in this study. A plot of these results, from the bi-objective case where volume and displacement are minimized, is presented in Fig. 10. In this figure, data points are coloured based on their associated concept and the optima for the three problem variants highlighted in green. The constraint for maximum displacement under a load of 1000 N for Lattice-1 was set at $\delta _{max} = 0.03$ mm, with Lattice-2 and Lattice-3 capturing the extreme points on the two objective axes. The best objective values in this data set, for each problem variant, is listed in Table 9.

**Table 9:** Best objectives for each problem variant, as reported by Parker et al.^10^.

	Lattice-1	Lattice-2	Lattice-3
$f(x^*)$	6.0991e + 03	2.7426e + 03	1.2965e − 03
Concept	Hex	Cube	Tkdh

The optimization code was written in Python, using the Optuna^36^ and Pymoo^31^ libraries, with lattice construction and FEA performed by CATIA V5-6R2017. The mesh sizes were determined automatically by CATIA’s Generative Structural Analysis workbench, allowing the software’s internal algorithms to select appropriate mesh sizes based on the geometric complexities and analysis requirements of each lattice structure. The CATIA models and macro scripts for the lattice structures can be found from the authors’ website (http://www.mdolab.net/Ray/Research-Data/LatticeApp.zip). The initial evaluations were set at $11D_{sum} + 1 = 100$, where $D_{sum}$ is the total sum of the number of variables, across all concepts. A total computing budget of $100 \times D_{max}$ evaluations was allocated, where $D_{max}$ is the maximum number of variables for a single concept. The multivariate TPE selected from a pool of 10,000 candidates.

Given the significant time required for the FEA simulation of each lattice structure—an average of 3 minutes per simulation, typically resulting in approximately 20 hours to evaluate 400 configurations—there are inherent limitations on the number of runs that can feasibly be conducted. This constraint not only restricts the overall number of simulation runs possible within a practical timeframe but also renders the detailed reporting of results for both the Believer and EI methods impractical. Consequently, we focus on presenting the results obtained using MvTPE across a total of five runs for each problem instance. The two previous studies provided a comparative analysis between MvTPE and the Believer and EI methods, highlighting their respective performances and behaviours. However, the aim of this study is to specifically explore the behavior of MvTPE when applied to real-world FEA problems, demonstrating its ability to effectively search across design concepts, in scenarios characterized by limited computational resources.

The results presented in Table 10 indicate that MvTPE consistently selected the expected concept, with objective values that are in the vicinity of the target values in Table 9. The problem variant on which MvTPE performed best was Lattice-2. This is expected as it only involves the computation and modeling of the lattice volume—a third order polynomial—which has far less complexity than the highly non-linear FEA simulations required to compute the displacement under load.

**Table 10:** Results for multivariate TPE on three variants of the lattice structures problem.

	Lattice-1	Lattice-2	Lattice-3
Best	6.9616e + 03	2.7505e + 03	1.8330e − 03
Worst	8.3681e + 03	2.7886e + 03	2.1040e − 03
Median	7.9110e + 03	2.7604e + 03	1.9440e − 03
Mean	7.7426e + 03	2.7672e + 03	1.9598e − 03
Std. dev	4.9753e + 02	1.4542e + 01	9.0744e − 05
Concept	Hex	Cube	Tkdh

Given are the best, worst, median and mean and standard deviation over 5 runs, along with the best concept for each problem variant.

**Figure 11:**
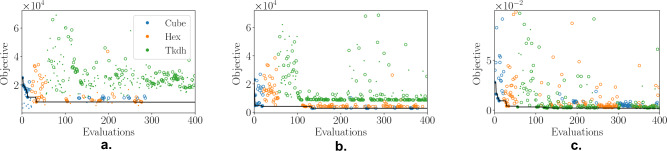
Plots for median runs of (**a**) Lattice-1, (**b**) Lattice-2 and (**c**) Lattice-3 problem variants. Circles indicate feasible solutions and small dots indicate infeasible ones. A circle is filled if it represents a solution improving on all prior to it.

Figure 11 provides the plots of the median runs for each of the three problem variants. As with Fig. 3d, these plots demonstrate that MvTPE explores the concept space well throughout the entire search, not myopically pursuing the first promising concept it encounters.

## Conclusions and future work

Concept design and selection is a critical step in the engineering design process and is known to have significant impact on the downstream life-cycle costs. It requires a balanced exploration of design spaces across multiple concepts, each with its own set of variables, to identify the best one(s) to proceed with. The inability of existing optimization methods to search across concepts severely limits their use in this early phase, since it may not be practical to run extensive optimization for each concept due to prohibitive computational/temporal/financial cost. To address this gap, in this study, we presented a framework for multi-concept optimization. The embedded methods include GP and RF models, used with believer- and EI-based acquisition functions, as well as a TPE-based sampler. These methods have been chosen to solve some of the key underlying challenges in dealing with such problems, such as efficient allocation of computing resources across concepts, handling of multiple variable types, and dealing with the limited number of function evaluations available when considering computationally expensive analyses. To the best of authors’ knowledge, this is the first study where these methods have been extended to deal with multi-concept design optimization problems. Numerical experiments on three practical case studies demonstrate the behavior of the underlying methods. Of particular significance is the TPE-based sampler which demonstrated its ability to efficiently explore multiple search spaces, across multiple concepts, and identified designs which were close to the ground truth. While in this study we have restricted the scope to single-objective optimization problems, the methods can be further enhanced to deal with multi-objective problems by integrating appropriate acquisition functions and ranking schemes in future work.

## Data Availability

All data generated or analysed during this study are included in this article and its supplementary information files, and the cited references. The codes are available upon request from the authors for research purposes.
